# The mediating effect of alexithymia between Avoidant/Restrictive Food Intake Disorder (ARFID) and suicidal ideation among a sample of Lebanese adults

**DOI:** 10.1371/journal.pone.0340095

**Published:** 2026-01-16

**Authors:** Lea Abou Nader, Georgio Chammas, Michael Chammas, Feten Fekih-Romdhane, Souheil Hallit, Sahar Obeid

**Affiliations:** 1 School of Medicine and Medical Sciences, Holy Spirit University of Kaslik, Jounieh, Lebanon; 2 School of Medicine and Medical Sciences, Saint Joseph University, Beirut, Lebanon; 3 Department of psychiatry “Ibn Omrane”, Razi hospital, The Tunisian Center of Early Intervention in Psychosis, Manouba, Tunisia; 4 Faculty of Medicine of Tunis, Tunis El Manar University, Tunis, Tunisia; 5 Applied Science Research Center, Applied Science Private University, Amman, Jordan; 6 Department of Psychology and Education, School of Arts and Sciences, Lebanese American University, Jbeil, Lebanon; European University of Rome, ITALY

## Abstract

**Background:**

Avoidant/Restrictive Food Intake Disorder (ARFID) is an underdiagnosed eating disorder consisting of food avoidance that is not related to body image concerns. New evidence suggests that individuals identified as having ARFID experience elevated suicidal ideations. This association could be mediated by alexithymia, described as having difficulty in recognizing or verbalizing emotions. Therefore, this study aimed to determine whether alexithymia mediates the link between ARFID symptoms and suicidal ideation among Lebanese adults.

**Methods:**

Between September and December 2024, a cross-sectional study was conducted using snowball sampling technique. 396 Lebanese adults (mean age = 26.26) took an online Arabic survey.

**Results:**

40.7% of the participants had thoughts of suicide. Younger age, being unmarried, less physical activity, higher ARFID scores, and increased alexithymia were significantly associated with suicidal ideation. Alexithymia fully mediated the link between ARFID symptoms and suicidal ideation (indirect effect: Beta = 0.02; Boot SE = 0.01; Boot 95% CI 0.01, 0.04). There was a strong correlation between higher avoidant/restrictive food intake disorder and higher alexithymia. There was also a significant association between higher alexithymia and the presence of suicidal ideation. Finally, there was no association between avoidant/restrictive food intake disorder and suicidal ideation.

**Conclusion:**

Our findings support alexithymia as a fundamental psychological process linking ARFID symptoms and suicidal ideation. Given the sociocultural stigma and limited mental health resources in Lebanon, clinicians should consider screening for alexithymia among individuals with eating disorders and suicidal thinking. Interventions that focus on emotions could therefore be used to reduce suicide risk in this vulnerable population.

## Introduction

Suicide is one of the biggest burdens affecting countries all around the world. Once considered taboo, people have been evoking this subject more and more throughout the years, hoping to spread awareness globally [1]. Based on the World Health Organization (WHO) reports, the third most common cause of death for those aged 15–29 is suicide, costing the lives of over 720 000 individuals annually [2]. However, in discussions surrounding this topic, individuals often overlook another dimension of this complex issue, which is suicidal ideation. According to the Centers for Disease Control and Prevention in the United States, suicidal ideation is defined as “thoughts of engaging in suicide-related behavior” [3]. Since ideation simply refers to thoughts about death and not the actual attempt, it is typically viewed as less dangerous than suicidal behavior itself [4]. Yet, Jobes and Joiner explained that suicidal ideation is equally significant and should not be taken lightly [5]. Even though suicide and suicidal ideation are not widely investigated in the Arab world due to a lack of reporting and overall underestimation [6], a study was conducted in 2023 on five Arab countries to assess the prevalence of suicidality among various demographic groups, using data collected from individuals who completed a free online questionnaire about depression and suicidality. This study found a high risk of suicidal ideation among young Arabic-speaking adults, particularly those under the age of 40 [7].

### ARFID with suicidal ideation

Nowadays, many factors may lead to this type of ideation, like internet addiction [8], lack of parental understanding [9], alcohol use disorder, child abuse [10], depression and bullying [11], as well as eating disorders [12]. Suicidal thoughts occur in about 25% to 30% of people with eating disorders [4,13,14,15], one of them being the Avoidant/Restrictive Food Intake Disorder (ARFID) [16,17]. According to the Diagnostic and Statistical Manual of Mental Disorders (DSM-5-TR) [18], ARFID is defined based on five criteria: first, dietary restriction or avoidance, which can be brought on by a loss of appetite, an unpleasant food experience, or even a fear of choking or vomiting after eating. Second, there is a persistent difficulty in achieving nutritional needs due to limitations in the quantity or variety of foods consumed. Third, the eating disorder is not attributable to food scarcity or culturally accepted practices. Fourth, neither body weight nor form perception is distorted, and the disorder is not specific to bulimia nervosa or anorexia nervosa. Fifth, the symptoms are not explained by a medical condition or another psychiatric disorder [18]. Several studies have shown a strong correlation between ARFID symptoms and suicidal ideation [4,16,19]. One of them conducted among a youth population showed that adolescents presenting with acute ARFID symptoms had a higher risk of suicidal ideas and self-harm compared to those with chronic onset [20]. Another research study that tackled the subject among adults found that in a group of 3299 adults diagnosed with ARFID, 22.9% had suicidal ideation [17]. These outcomes can be attributable to several reasons, including frustration and hopelessness due to problems in the identification of the diagnosis of ARFID itself and the complications related to the treatment [21], or even social isolation and loneliness that may accompany the daily life of people with ARFID symptoms [22].

### ARFID and alexithymia

To understand even more the relationship between ARFID and suicidal ideation, one psychological trait may play a central role in explaining this relationship, which is alexithymia. Alexithymia is defined as a personality trait characterized by difficulty identifying, describing, and understanding one’s own emotions, as well as trouble expressing emotions to others [23]. Alexithymia has been associated with a variety of psychiatric diseases, one of them being eating disorders [24]. More so, it has been linked to emotional difficulties in individuals with eating disorders and has been an underlying cause of the development and the maintenance of these disorders [25,26]. Most investigations have reported greater degrees of alexithymia in those with eating disorders and disrupted eating than in healthy controls [23], especially people having ARFID [27]. This may be due to a partial decrease in interoception and emotional awareness. In fact, people with ARFID typically find it difficult to identify hunger or satiety signals, which would reflect difficulties processing and expressing emotional states [28]. Moreover, studies have indicated that alexithymia is not only more common in this type of person, but it may also predict more severe clinical manifestations and worse treatment results [29].

### Suicidal ideation and alexithymia

Furthermore, alexithymia is strongly correlated with a higher risk of suicidal ideation. In fact, due to the inability of these individuals to identify or express emotional distress, they tend to internalize their suffering, thus increasing the risk of depressive symptoms and suicidal thinking [30,31]. Moreover, people with high degrees of alexithymia may find it challenging to seek treatment, build meaningful emotional relationships, or even find solutions to control negative effects, all of which are crucial defensive mechanisms against suicide [31]. Several studies have identified alexithymia as a significant predictor of suicidal ideation regardless of the presence of depression and anxiety, suggesting that it contributes uniquely to suicidal vulnerability [32]. For example, De Berardis et al. (2008) investigated psychiatric outpatients with major depressive disorder and discovered that alexithymia was highly associated with suicidal ideation, even after controlling for levels of depression and anxiety. According to the authors, people with alexithymia may lack the cognitive skills required to manage emotional pain, making them more inclined to express their distress through self-harm or suicidal ideation [31]. Similarly, Hintikka et al. (2004) conducted a 12-month follow-up study in a general population sample, and found that people with high alexithymia scores had consistently greater rates of suicidal thoughts than those with low alexithymia, regardless of their baseline depressive state [32].

### The present study

Taking all these findings, we can suggest that alexithymia would be a mediating factor in the relation between ARFID symptoms and suicidal thoughts. Individuals with ARFID symptoms would be more inclined to have suicidal thoughts [17], not only due to the problems that come with this type of eating disorder, but also due to their difficulties with emotional perception and regulation [28,33]. Having said that, this mediational process has not been fully examined in past research. While many studies have independently explored the associations among ARFID and suicidal ideation [4,16,17], ARFID and alexithymia [34,35], and alexithymia and suicidal ideation [31,32,36,37], few, if any, have combined these variables within one model to assess their interdependence.

This knowledge gap is particularly present in Lebanon, a country that has been facing major socioeconomic breakdowns, political crisis, and collective trauma, especially during the past few years [38]. All these events have aggravated the population’s mental health issues, especially among youth and vulnerable groups [38]. Yet, ignorance, limited access to treatment and cultural barriers continue to delay the early diagnosis and treatment of psychiatric conditions like ARFID. Until now, ARFID has been underdiagnosed and understudied in Lebanon, plus people are poorly familiar with concurrent psychological traits like alexithymia, which makes it a challenge to study these variables among the Lebanese population. Given these factors, it is necessary to investigate the mechanisms by which underlying affective traits might contribute to risk for suicidal ideation in people with eating disorders. Clarifying the role of alexithymia in a population known to have eating disorders like ARFID [39] and suicidal thoughts [9], might be essential for mental health screening, intervention planning, and sensitive program development. Therefore, the objective of this study is to examine the mediating effect of alexithymia between ARFID and suicidal ideation in the Lebanese population.

## Methods

### Ethics approval and consent to participate

This study received ethical approval from the ethics committee at Notre Dame des Secours University Hospital. All participants gave their informed consent, with online submission of the form considered equivalent to written consent. All procedures followed relevant guidelines and regulations, in line with the Declaration of Helsinki.

### Study design and participants

This cross-sectional study was conducted between September and December 2024, using a snowball sampling technique, where existing participants recruited future participants from social work and contacts [40]. Using Google Forms, we created a survey and distributed it via social media and messaging platforms like WhatsApp, Instagram, and Messenger to Lebanese adults from a variety of backgrounds, including university students and other community members. Every respondent took part in the poll freely and without receiving compensation. Every respondent took part in the study voluntarily and without compensation. Participation was open to all individuals over the age of 18. Individuals who fully declined to participate and those younger than 18 years old were excluded from the study, as well as pregnant women because pregnancy alters a woman’s appetite, metabolism, nutrient requirements, and eating patterns to support fetal development [41].

### Minimal sample size calculation

Following Fritz and MacKinnon’s formula [42] $n=\frac{L}{f\text{2}}+k+\text{1}$, where f = 0.26 for a moderate effect size, L = 7.85 for an α error = 5%, power β = 80%, and k = 10 variables included in the mediation model, a minimum sample size of 127 was performed a priori to guarantee sufficient statistical power.

### Questionnaire

The average time to complete the Arabic questionnaire was 15 minutes. The first section consisted of sociodemographic information such as age, gender, marital status, and the Household Crowding Index (HCI), which is calculated by dividing the number of household members, minus newborns, by the number of rooms in the house, minus the kitchen and bathrooms [43]. Whenever we get a high household crowding index, it indicates that the family has a low socioeconomic status. The physical activity index was calculated by multiplying the intensity, frequency, and duration of physical activity [44]. Financial burden is assessed using the single-item financial stress scale. This scale is based on the following question: “How stressed do you feel about your personal finances in general?”. Responses are rated on a 10-point scale ranging from 1 (“Overwhelming Stress”) to 10 (“No Stress at All”) [45]. The second section included the scales listed below.

Nine Item Avoidant/Restrictive Food Intake disorder screen (NIAS). The NIAS is designed to screen for ARFID and was validated in Arabic, especially in Lebanon [46]. It consists of 9 items which are scored on a 6-point Likert scale, “Strongly disagree,” “Disagree,” “Slightly disagree,” “Slightly agree,” “Agree,” and “Strongly agree” [47]. It includes three subscales composed of three items each as follows: Picky eating, Appetite, and Fear. More avoidant/restrictive eating is indicated by greater scores. It has been suggested to use cutoff values of ≥ 10, ≥ 9, and/or ≥ 10 to identify those who meet the NIAS dimensions Picky eating, Appetite, and Fear, respectively [48] (Cronbach’s α in this study = 0.87).

The Columbia-Suicide Severity Rating Scale (C-SSRS). Researchers from the universities of Columbia, Pennsylvania, and Pittsburgh created the 10-item C-SSRS, a dichotomous scale, to assess suicidal behavior (5 questions) and ideation (5 questions) [49]. It was validated in Arabic, especially in Lebanon [50]. The five questions used in this study to tackle suicidal ideation were: “wish to be dead”, “suicidal thoughts”, “suicidal thoughts with a method”, “suicidal intent”, and “suicidal intent with a specific plan”. Each item was coded as No = 0 and Yes = 1, and responses were summed to yield a total suicidal ideation score ranging from 0 to 5, with higher scores indicating greater severity of suicidal ideation. A total score of 0 indicated no suicidal ideation [51]. In the original Arabic validation, the suicidal ideation subscale showed good internal consistency (αCronbach = 0.796) [50]; in the current sample, the internal reliability remained high (Cronbach’s α in this study = 0.81).

Toronto Alexithymia Scale (TAS-20). The TAS used to measure alexithymia [52,53] was validated in Lebanon [54]. It is graded on a 5-point Likert scale (1 = strongly disagree to 5 = strongly agree), with higher scores reflecting more alexithymia. It is divided into three sub-scales: Difficulty Identifying Feelings (comprising 7 items), Difficulty Describing Feelings (comprising 5 items), and Externally Oriented Thinking (comprising 8 items) (Cronbach’s α in this study = 0.84).

### Statistical analysis

Data analysis was performed using SPSS software version 27. The suicidal ideation score and its LOG transformation did not follow a normal distribution. As a result, the original score was converted into a binary variable indicating the presence or absence of suicidal ideation. To compare means between two groups, the Student t-test was used, while the Chi-square test was applied for categorical variables. Mediation analysis was conducted using the PROCESS Macro, version 4.2, Model 4, specifying a logistic regression framework for the binary dependent. The analysis used 5,000 bootstrap samples and bias-corrected 95% confidence intervals to estimate indirect effects. Prior to running the mediation model, multicollinearity was assessed using the variance inflation factor (VIF) statistics for all predictors and covariates. All VIF values were < 5, indicating no multicollinearity. This analysis produced three paths: path A (independent variable to mediator), path B (mediator to dependent variable), and path C’ (direct effect of the independent variable on the dependent variable). Mediation is considered significant if the confidence interval does not include zero [55]. Covariates included in the model were those with a p-value < 0.250 in the bivariate analysis. A p-value < 0.05 was considered statistically significant.

## Results

### Participants

A total of 396 participants completed the questionnaire. The mean age was 26.26 years, and 26.5% of the participants were females. All participant characteristics can be found in the table below (Table 1).

**Table 1:** Sociodemographic and other characteristics of participants (N = 396).

Variable	N (%)		
**Gender**			
Male	291 (73.5%)		
Female	105 (26.5%)		
**Social status**			
Single, divorced, widowed	326 (82.3%)		
Married	70 (17.7%)		
**Suicidal ideation**			
No	235 (59.3%)		
Yes	161 (40.7%)		
	**Mean ± SD**	**Minimum**	**Maximum**
**Age (years)**	26.26 ± 8.18	18	60
**Household crowding index**	1.00 ± 0.55	0.06	4
**Financial burden**	5.47 ± 2.60	1	10
**Physical activity**	28.10 ± 22.15	1	100
**Avoidant/Restrictive Food Intake Disorder**	13.76 ± 9.85	0	45
**Suicide Severity Rating Scale Score**	0.97 ± 1.55	0	6
**Alexithymia**	55.19 ± 12.73	23	91

### Bivariate analysis

A higher percentage of people with suicidal ideation was found in single, divorced, widowed vs married. Additionally, a younger mean age, a lower mean physical activity level, and a higher mean avoidant/restrictive food intake disorder and alexithymia were significantly found in participants who had suicidal ideation (Table 2).

**Table 2:** Bivariate analysis of factors associated with suicidal ideation.

Variable	Absence of suicidal ideation	Presence of suicidal ideation	P	Effect size
**Gender**			0.187	0.066
Male	167 (57.4%)	124 (42.6%)		
Female	68 (64.8%)	37 (35.2%)		
**Social status**			**0.011**	0.127
Single, divorced, widowed	184 (56.4%)	142 (43.6%)		
Married	51 (72.9%)	19 (27.1%)		
	**Mean ± SD**		**P**	**Effect size**
**Age (years)**	27.13 ± 8.65	24.98 ± 7.29	**0.008**	0.265
**Household crowding index**	0.96 ± 0.56	1.05 ± 0.55	0.100	0.169
**Financial burden**	5.28 ± 2.51	5.76 ± 2.69	0.072	0.184
**Physical activity**	30.66 ± 23.16	24.35 ± 20.08	**0.004**	0.288
**Avoidant/Restrictive Food Intake Disorder**	12.76 ± 9.98	15.21 ± 9.50	**0.015**	0.250
**Alexithymia**	51.36 ± 12.17	60.80 ± 11.42	**<0.001**	0.795

Numbers in bold indicate significant p values.

### Analysis of mediation with the presence/absence of suicidal ideation considered as the dependent variable

**Fig 1:**
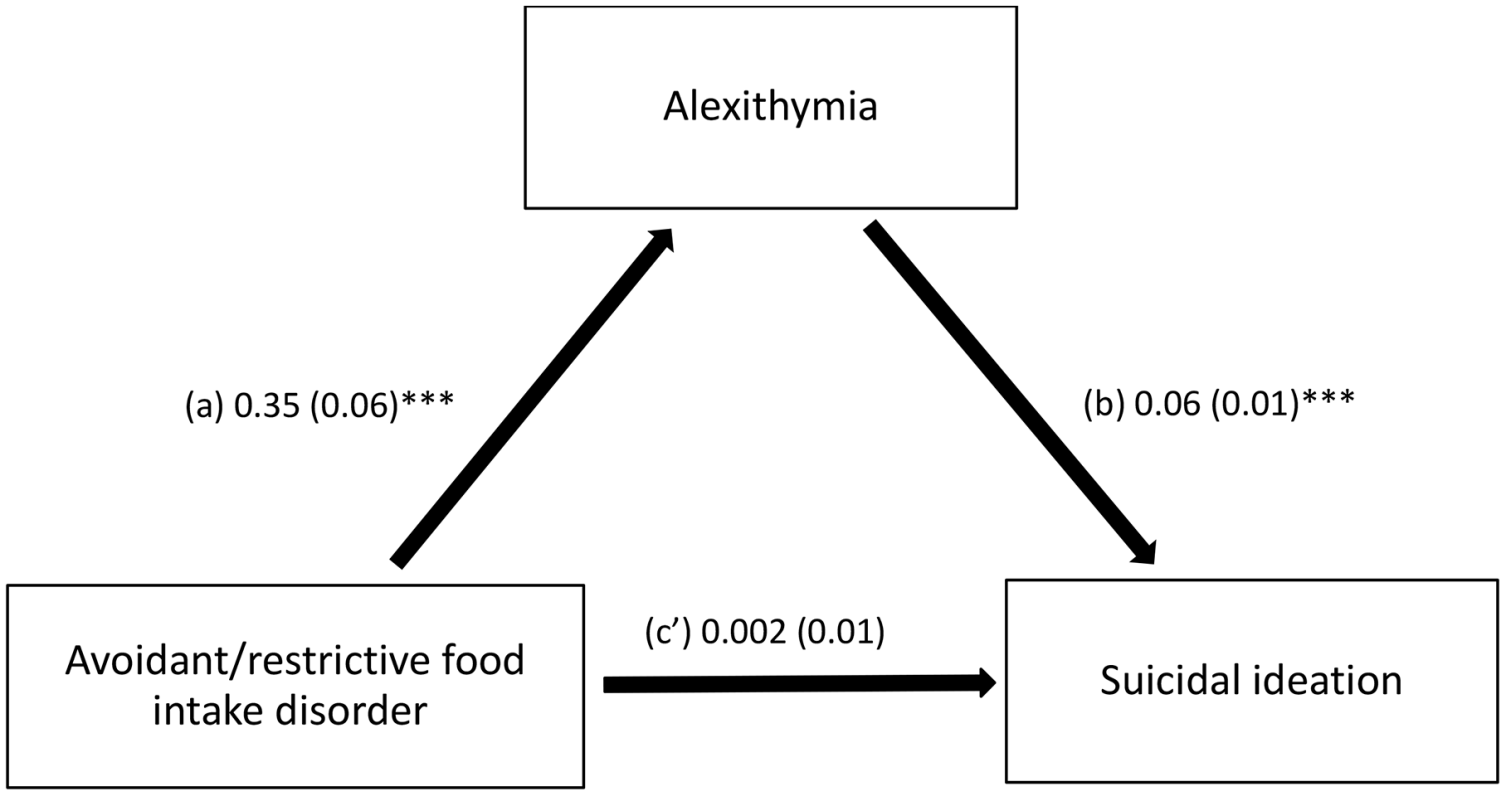
Mediation effect of alexithymia between avoidant/restrictive food intake disorder and suicidal ideation. (a) Relation between avoidant/restrictive food intake disorder and alexithymia (R^2^ = 0.150); (b) Relation between alexithymia and suicidal ideation (R^2^ = 0.226); (c’) Direct effect between avoidant/restrictive food intake disorder and suicidal ideation (R^2^ = 0.226). ***p < .001.

The results of the mediation analysis were adjusted over the following variables: gender, social status, age, household crowding index, financial burden and physical activity. Alexithymia fully mediated the association between avoidant/restrictive food intake disorder and suicidal ideation (indirect effect: Beta = 0.02; Boot SE = 0.01; Boot 95% CI 0.01, 0.04). Higher avoidant/restrictive food intake disorder was strongly linked to higher levels of alexithymia, which in turn increased the odds of suicidal ideation. Finally, there was no significant association between avoidant/restrictive food intake disorder and suicidal ideation (Fig 1; Table 3). The logistic regression model explained 22.6% of the variance (Nagelkerke R^2^ = 0.226) of suicidal ideation.

**Table 3:** Mediation model results with suicidal ideation as a binary outcome.

Path	Relationship	Coefficient	SE	*p*	95% CI
a	ARFID **→** Alexithymia	0.35	0.06	<0.001	0.23-0.47
b	Alexithymia **→** Suicidal ideation	0.06	0.01	<0.001	0.04-0.08
c’	ARFID **→** suicidal ideation (direct effect)	0.002	0.01	0.884	−0.002; 0.003
ab	Indirect effect	0.02	0.01	–	0.01; 0.04

SE = Standard error; ARFID = Avoidant Restrictive Food Intake Disorder; CI = Confidence Interval.

## Discussion

The study aimed to explore the mediating role of alexithymia in the relationship between Avoidant/Restrictive Food Intake Disorder and suicidal ideation among Lebanese adults. The results demonstrated that alexithymia significantly mediated the link between ARFID symptoms and suicidal ideation. Hence, higher avoidant/restrictive food intake disorder was significantly associated with higher alexithymia, and higher alexithymia was significantly associated with the presence of suicidal ideation. Finally, avoidant/restrictive food intake disorder was not highly linked with suicidal ideation.

### ARFID and alexithymia

Consistent with prior research, we found in our study that adults with ARFID symptoms were associated to higher level of alexithymia. This correlates with Becker et al. (2024) who reported higher emotion regulation difficulties in adults with ARFID symptoms compared to non-clinical participants [56]. Moreover, meta-analytic studies have proven that people diagnosed with eating disorders consistently scored higher on alexithymia scales compared to healthy people [57]. This can be explained by the lack of understanding or labeling of their own emotions, which are highly associated with ARFID features [57].

Furthermore, this increased level of alexithymia among people with ARFID symptoms can be explained by impaired interoceptive cognition, which is a diminished ability to interpret body’s sensations like hunger or satiety [58]. These people often fail to distinguish between physical sensations and emotional distress, which aggravates their food restriction trait [23,28]. Moreover, studies done on the Lebanese adult population showed that a high level of alexithymia was associated with psychological distress, emotional regulation difficulties, and insecure attachment, which are factors that can interact with ARFID features [46,54]. These findings highlight a close link between alexithymia and the restrictive behaviors characteristic of ARFID.

### Alexithymia and suicidal ideation

Our findings showed that higher alexithymia scores were associated with a greater likelihood of suicidal ideation. This is in line with other studies, like a meta-analysis done in 2019, which revealed a strong association (r ≈ 0.54) between alexithymia and suicidal thoughts, and a weaker link to actual suicidal attempts [36]. This failure to recognize one’s emotions in alexithymia aggravates one’s inner self-harm, depression, and suicidal ideation [59]. Thus, these people lack adaptive coping skills such as talking to others, sharing their thoughts, or seeking professional help, making them more likely to suppress their feelings and withdraw from any social interaction, which worsens suicidal thinking [32].

Furthermore, a national cross-sectional survey that enrolled 789 Lebanese adults found that low self-esteem and alexithymia were associated with a higher level of suicidal thoughts [50,60]. In fact, the Lebanese population has been facing years of political instability, economic collapse, and social insecurity, which altogether can lead to emotional suppression and alexithymia [61,62]. Consequently, the combination of high levels of alexithymia and widespread socioeconomic suffering may contribute to the rising incidence of suicidal thoughts in Lebanon [50,61,62].

### Mediating role of alexithymia

Our results also revealed that alexithymia mediated the association between ARFID symptoms and suicidal ideation. This implies that alexithymia, a marker of emotional dysregulation [63], may be the critical bridge by which ARFID symptoms influence suicidality. Our mediation results coincide with theoretical and empirical theories that suggest that suicidal thoughts accompanying ARFID symptoms are deeply linked to an underlying failure to recognize and communicate emotions [64]. The idea that alexithymia impairs the ability to identify internal emotional states like frustration, fear, or loneliness can explain food restriction or avoidance [23,65]. Plus, in addition to maintaining disordered eating, it will increase the likelihood of suicidal thoughts by cultivating a sense of hopelessness [66]. Altogether, these findings support the idea that alexithymia is a key factor in the relationship between ARFID symptoms and suicidal thoughts, where the inability to process internal emotions is highly associated to more severe ARFID symptoms and the emergence of suicidal thoughts [67].

### Sociodemographic variables

In the present study, a higher percentage of people with suicidal ideation was found in single, divorced, and widowed individuals in comparison to married individuals. Additionally, a younger mean age was more prone to have suicidal ideation. Several studies revealed that younger individuals and unmarried participants reported significantly higher levels of suicidal ideation [68,69,70]. This can be due to poor problem-solving skills and impulsiveness among the younger population [71], as well as reduced social support or belonging and feelings of loneliness among single individuals [72].

Moreover, a lower physical activity level was found in participants who had suicidal ideation. Indeed, physical activity is known to enhance mood, reduce stress, and improve cognitive flexibility, all of which are necessary to avoid suicidal ideation [73]. For example, Vancampfort et al. (2018) demonstrated through a large-scale meta-analysis that physical inactivity was linked to a higher risk of suicidal thoughts, particularly in youth and young adults across global populations [74]. In fact, regular physical activity is known to modify neurotransmitter release inside the brain, like increasing serotonin and endorphin levels, which are responsible for feelings of high esteem, increasing social engagement, and reducing stress [75]. That’s why, in people with low physical activity, the levels of these neurotransmitters will be lower, thus putting the individual at risk for isolation and suicidal ideation [76].

### Clinical implications

By demonstrating that alexithymia mediates the relation between ARFID symptoms and suicidal ideation, this research validates the necessity of incorporating emotional assessment and regulation into intervention strategies and screening programs. Clinicians need to understand that individuals presenting with ARFID symptoms not only struggle with eating behaviors but are more likely to have difficulties expressing and identifying their emotions, thus increasing their risk of suicidal thoughts.

In Lebanon, mental health is often seen as taboo, and access to care is limited due to socioeconomic barriers. That’s why our study is critical to find solutions for routine screening for alexithymia while dealing with people with ARFID symptoms having suicidal thoughts, plus to come up with therapeutic interventions that incorporate emotion-focused therapy or dialectical behavior therapy so that physicians can target emotional processing skills.

Finally, given the high rates of psychological distress in our country, early identification of alexithymia may serve as a preventive tool to reduce the risk of suicidality among the vulnerable Lebanese individuals. Nutritional rehabilitation, psychoeducation, and emotional skills training are the keys to improving clinical outcomes and suicide risk in patients with ARFID symptoms.

### Limitations

This study presents some limitations. First, the study relied on self-reported questionnaires to assess ARFID, alexithymia and suicidal ideations. Given the sensitivity of these constructs, the use of self-report measures may introduce social desirability or underreporting, which could affect the accuracy of participants’ responses. it was based on self-reported questionnaires to assess suicidal thoughts and alexithymia, which may lead to social desirability bias. Second, this study is a cross-sectional type, and hence it is not possible to draw causal relationships between the studied variables. Future longitudinal research is needed to confirm whether alexithymia truly mediates the pathway from ARFID symptoms to suicidal ideation. Moreover, prospective studies could help determine whether different ARFID subtypes are differentially associated with suicidal risk. Third, the responses were collected based on a snowball technique, which is a type of selection bias and may not be representative of the whole population, with certain demographic or psychological characteristics being overrepresented. Fourth, the disproportionate gender representation may introduce sampling bias, potentially limiting the generalizability of the findings, given that gender differences are known to influence both alexithymia levels and suicidal ideation patterns. Future studies should evaluate the different nuances considering ARFID subtypes to see if alexithymia may have a different effect according to the type of ARFID.

## Conclusion

In conclusion, this study offers strong evidence that alexithymia fully mediates the relation between ARFID symptoms and suicidal ideation. These results prove that emotional difficulties should be taken into consideration while working with individuals with ARFID symptoms before they develop suicidal thoughts. Strategies for suicide prevention and early intervention may focus on treating alexithymia in people with ARFID. Moreover, public awareness should be raised concerning alexithymia and its impact on the vulnerable population, especially in Lebanon. Finally, Lebanese physicians and researchers should use this study as a starting point for future research on ARFID, alexithymia, and suicidal ideation. Future studies should explore targeted therapeutic interventions that aim to enhance emotional awareness and regulation in ARFID patients. Longitudinal research also needs to be undertaken to assess the long-term effects of such intervention and suicide risk reduction.
